# Normofractionated stereotactic radiotherapy versus CyberKnife-based hypofractionation in skull base meningioma: a German and Italian pooled cohort analysis

**DOI:** 10.1186/s13014-019-1397-7

**Published:** 2019-11-12

**Authors:** Conti Alfredo, Senger Carolin, Acker Güliz, Kluge Anne, Pontoriero Antonio, Cacciola Alberto, Pergolizzi Stefano, Germanò Antonino, Badakhshi Harun, Kufeld Markus, Meinert Franziska, Nguyen Phuong, Loebel Franziska, Vajkoczy Peter, Budach Volker, Kaul David

**Affiliations:** 1 Department of Neurosurgery, University of Bologna, Bologna, Italy; 2 Department of Neurosurgery, Charité Universitätsmedizin Berlin, Berlin, Germany; 3 CyberKnife Center, Charité Universitätsmedizin Berlin, Berlin, Germany; 4 Department of Radiation Oncology, Charité Universitätsmedizin Berlin, Berlin, Germany; 5 Berlin Institute of Health (BIH), 10178 Berlin, Germany; 6 Department of Radiation Oncology, University of Messina, Messina, Italy; 7 Ernst von Bergmann Medical Center, Department of Radiation Oncology, Potsdam, Germany

**Keywords:** Meningioma, Skull Base, Radiosurgery, Fractionated stereotactic radiotherapy, Hypofractionated stereotactic radiotherapy, CyberKnife

## Abstract

**Background:**

This retrospective German and Italian multicenter analysis aimed to compare the role of normofractionated stereotactic radiotherapy (nFSRT) to CyberKnife-based hypofractionated stereotactic radiotherapy (CK-hFSRT) for skull base meningiomas.

**Methods:**

Overall, 341 patients across three centers were treated with either nFSRT or CK-hFSRT for skull base meningioma. Treatment planning was based on computed tomography (CT) and magnetic resonance imaging (MRI) following institutional guidelines. Most nFSRT patients received 33 × 1.8 Gy, and most CK-hFSRT patients received 5 × 5 Gy. The median follow-up time was 36 months (range: 1–232 months).

**Results:**

In the CK-hFSRT group, the 1-, 3-, and 10-year local control (LC) rates were 99.4, 96.8, and 80.3%, respectively. In the nFSRT group, the 1-, 3-, and 10-year LC rates were 100, 99, and 79.1%, respectively. There were no significant differences in LC rates between the nFSRT and CK-hFSRT groups (*p* = 0.56, hazard ratio = 0.76, 95% confidence interval, 0.3–1.9). In the CK-hFSRT group, only one case (0.49%) of severe toxicity (CTCAE 4.0 **≥** 3) was observed. In the nFSRT group, three cases (2.1%) of grade III toxicity were observed.

**Conclusion:**

This analysis of pooled data from three centers showed excellent LC and low side effect rates for patients treated with CK-hFSRT or nFSRT. The efficacy, safety, and convenience of a shortened treatment period provide a compelling case for the use of CK-hFSRT in patients with moderate size skull base meningioma and provided that OAR constraints are met.

## Background

Treating skull base meningioma using traditional surgical methods is difficult because of its close proximity to critical structures such as the cranial nerves, brainstem, and major blood vessels (including the cavernous sinus and carotid arteries). Historically, a surgical approach with the aim of gross total resection (GTR) was considered the gold standard in symptomatic patients. Arguments in favor of the surgical approach include instant reduction of the mass effect as well as risk of false diagnosis of tumor type and grade in patients without surgery where no tissues were obtained. However, complete surgical removal is often difficult to achieve without compromising the intricate neuroanatomic structure of the skull base and the risk of local relapse following incomplete resection without adjuvant treatment is high. Also, morbidity following surgery is high especially in the elderly patients where there is a significant rate of post-surgery mortality [1, 2].

Radiotherapy (RT) is a potential alternative for definitive treatment, and plays a role in the adjuvant situation after incomplete resection of grade I meningiomas as well as after complete or incomplete resection of grade II and III meningiomas. Traditional radiotherapeutic approaches consisted of normofractionated stereotactic RT (nFSRT) but recent availability of high-precision devices including the CyberKnife and Gamma Knife in first world have resulted in both hypofractionated stereotactic radiotherapy (hFSRT) and radiosurgery (SRS) to become a fast, safe and convenient alternative approach [3–7].

Traditional nFSRT takes advantage of the differing cell repair potentials of tumor versus healthy tissue in an elegant fashion, by allowing for the healthy tissue to repair in the interval between two treatments it is possible to achieve a broad therapeutic window. On the other hand, in hFSRT and SRS treatment approaches higher single doses are applied, thus requirements for conformity and steepness of dose gradient are increased, especially if the treatment volume is in close proximity to organs at risk (OAR) [1, 8, 9].

This retrospective German and Italian multicenter pooled analysis aimed to evaluate the role of CyberKnife-based hFSRT in the treatment of skull base meningioma cases as compared to a normofractionated cheme of stereotactic irradiation.

## Methods

### Patient selection and tumor characteristics

We retrospectively reviewed our clinical databases, and collected data on patients treated between October 1995 and November 2018 across three institutions. Patients examined in this study were pooled from the Department of Radiation Oncology at [blinded], the CyberKnife Center at [blinded], and the CyberKnife Center at [blinded]. Patients were included if they had undergone radiotherapy of a skull base meningioma excluding those who received a single fraction stereotactic treatment. Diagnosis had to be made either histologically or through imaging-based approaches.

### Ethics

The study was approved by our local ethics committees in Italy and Germany.

### Stereotactic hypofractionated radiotherapy/radiosurgery at our centers in Germany and Italy

Treatments were delivered using the CyberKnife (Accuray Inc., Sunnyvale, CA), an image-guided, frameless, Linac-based, 6 MV radiosurgery system [5, 10, 11]. The patient’s head was immobilized with a thermoplastic mask during planning computed tomography (CT) and treatment. During treatment, patient positions were monitored with orthogonal x-ray images approximately every minute.

For planning purposes, a high-resolution contrast-enhanced thin-slice head CT was performed using a multi-slice CT scanner. Additionally, thin-section, contrast-enhanced, T1-weighted magnetic resonance imaging (MRI) was performed. Contouring of the tumor volume and the critical structures were performed on the co-registered MRI and CT dataset using the MultiPlan software (Accuray Inc., USA).

Manual contouring was done in the axial slices with simultaneous display of contours on reconstructed orthogonal images. The gross tumor volume (GTV) was defined as the tumor volume based on CT and MRI, and no safety margin was added. The selection of the marginal and maximal doses and the number of sessions were influenced by multiple factors including tumor volume, adjacency to organs at risk (optic nerve, chiasm, and brainstem) as well as the area that previously received irradiation. The ray-tracing algorithm was routinely used for nonisocentric beam delivery. The dose was prescribed to the median prescription isodose line covering the GTV was 75%. Dose constraints to organs at risk (OAR) for five fractions of CK-hFSRT were as follows: ≤0.2 cm^3^ of the optic pathway could receive 23.0 Gy with a maximum point dose of 25.0 Gy in ≤0.035 cm^3^ and ≤ 0.5 cm^3^ of the brainstem could receive 23.0 Gy with a maximum point dose of 31 Gy in ≤0.035 cm^3^ [12].

### Stereotactic radiotherapy at our department in Germany

From 1995 to 2003, meningioma patients underwent “sharp” fixation using a stereotactic head ring and an oral bite plate. A 6-MV Linac (Varian Medical Systems, USA) with an add-on micro multi-leaf collimator (BrainLAB, Germany) was used. Coordinates for SRS were set by a laser-based stereotactic localizer. This setup allowed delivering shaped beams. In 2004, the department started using NovalisTx with beam shaping capability using built-in MLC and image guidance with ExacTrac (Varian Medical Systems, USA and BrainLAB, Germany). The image-guided frameless system enabled us to image the patient at any couch position using a frameless positioning array. A three-dimensional treatment planning based on CT and co-registered MRI was done with Brainscan (BrainLAB, Germany), which was later replaced by iplanRT (BrainLAB, Germany). The GTV was defined as the area of contrast enhancement on T1-weighted MR images and the planning target volume (PTV) included a 1–2 mm isotropic safety margin. The dose was prescribed to a reference point, representing 100%. Patients received 95% of the prescribed dose at the PTV margin. Dose constraints for OAR were as follows: optic nerves, chiasma, and brainstem could receive maximal 54 Gy according to Quantitative Analyses of Normal Tissue Effects in the Clinic (QUANTEC) [13].

### Algorithms

Meningioma localization was classified according to the new CLASS algorithmic scale: In their publication Lee et al. classified tumor location based on the experience of the senior author. “Low-risk” locations included convexity and lateral skull base (lateral and middle sphenoid wing, posterior petrous). Olfactory groove, planum sphenoidale, tentorial (lateral/paramedian), parasagittal, intraventricular, cerebellopontine angle, falcine, posterior/lateral foramen magnum as well as para-sigmoid and para-transverse sinus locations constituted the “moderate risk” group. The “high-risk” locations included clinoidal, cavernous sinus, tuberculum sellae, tentorial (medial/incisural), ventral petrous, petroclival and anterior/anterolateral foramen magnum [14].. Meningiomas of the optical nerve sheath were not included in the original CLASS algorithmic scale, in the presented work they were classified as high-risk.

### Calculation of doses equivalence

Calculations of equivalent doses of radiation to normofractionation (EQD2) and biologically effective dose (BED) were based on a radiobiological model previously described [5]. In brief, we analyzed the clinical data available in the literature on meningioma irradiation. We then selected two different dose/fraction schemes that resulted in similar tumor control rates. According to our evaluations, 13 Gy in single fraction provides LC rates that are similar to 54 Gy in 30 fractions for meningiomas. Using the LQ model, it is then possible to calculate the α/β of meningiomas. Actually, if the BED of these two schedules of irradiation are equivalent, we can infer that the α/β of meningiomas is equal to 2 [5].

### Follow-up and data collection

All patients underwent serial radiological evaluations and, in selected cases, endocrinological and ophthalmological examinations. The first follow-up examination usually occurred 6 months after treatment. Radiological assessment consisted of contrast-enhanced T1-weighted MRI in all cases.

Patients were followed up after treatment at the three departments in 3–6-month intervals for the first year. From the second year on, follow-up intervals were extended to 6–12 months, or as required clinically. We included the latest available follow-up in this analysis. Progression-free survival (PFS as local control [LC]) was determined based on the respective radiologist’s judgement. Treatment of progressing tumors was discussed in multidisciplinary tumor boards.

### Statistical analysis

The patient and tumor characteristics were compared between the two treatment groups using the chi-squared test (categorical variables) or Mann–Whitney U test (continuous variables). The Kaplan–Meier method was used to calculate the PFS probabilities from the last day of RT. Univariate analyses was performed using Cox regression analysis. All statistical analyses were performed using SPSS version 24.0 (IBM Inc., Armonk, NY, USA). *P*-values of less than 0.05 were considered statistically significant for this study.

## Results

### Patient cohort

Patient characteristics are shown in Table 1. A total of 341 patients were enrolled in this study, 49 patients enrolled from [blinded], 136 patients enrolled from [blinded], and 156 patients enrolled from [blinded]. Median follow-up time to last contact or progress on MRI was 36 months (range 1–232 months). Two hundred and five patients received CK-hFSRT and 136 patients received nFSRT treatments. Compared with 41.9% of nFSRT patients, approximately two-thirds of CK-hFSRT patients received definitive radiotherapy at primary diagnosis (65.9%).

**Table 1:** Patient characteristics

	CK hFSRT	nFSRT
CKC (blinded)	49	0
CKC (blinded)	156	0
RT (blinded)	0	136
Age [y], median (range)	57 (27–86)	58 (20–84)
DOTATOC PET RT-planning	6 (2.9%)	6 (4.4%)
Total dose [Gy], median (range)	25 (5–61)	59.4 (32.4–63)
Class algorithm: Low risk group, n (%)	6 (3%)	2 (1.5%)
Class algorithm Intermediate risk group, n (%)	12 (5.9%)	10 (7.4%)
Class algorithm High risk group, n (%)	184 (91.1%)	124 (91.2%)
Median follow-up	32.5 (2–135)	41.5 (1–232)
Definitive radiotherapy at first diagnosis	135 (65.9%)	57 (41.9%)
Adjuvant radiotherapy at first diagnosis	16 (7.8%)	34 (25%)
Definitive radiotherapy at relapse	54 (26.3%)	45 (33.1%)
No surgery	135 (65.9%)	52 (38.2%)
Biopsy	6 (2.9%)	5 (3.7%)
Subtotal resection	37 (18.0%)	52 (38.2%)
Gross total resection	25 (12.2%)	18 (13.2%)
Resectional status unclear	2 (1.0%)	9 (6.6%)
Previous RT	4 (2.0%)	1 (0.7%)

### Radiation doses and schedules

**Fig. 1:**
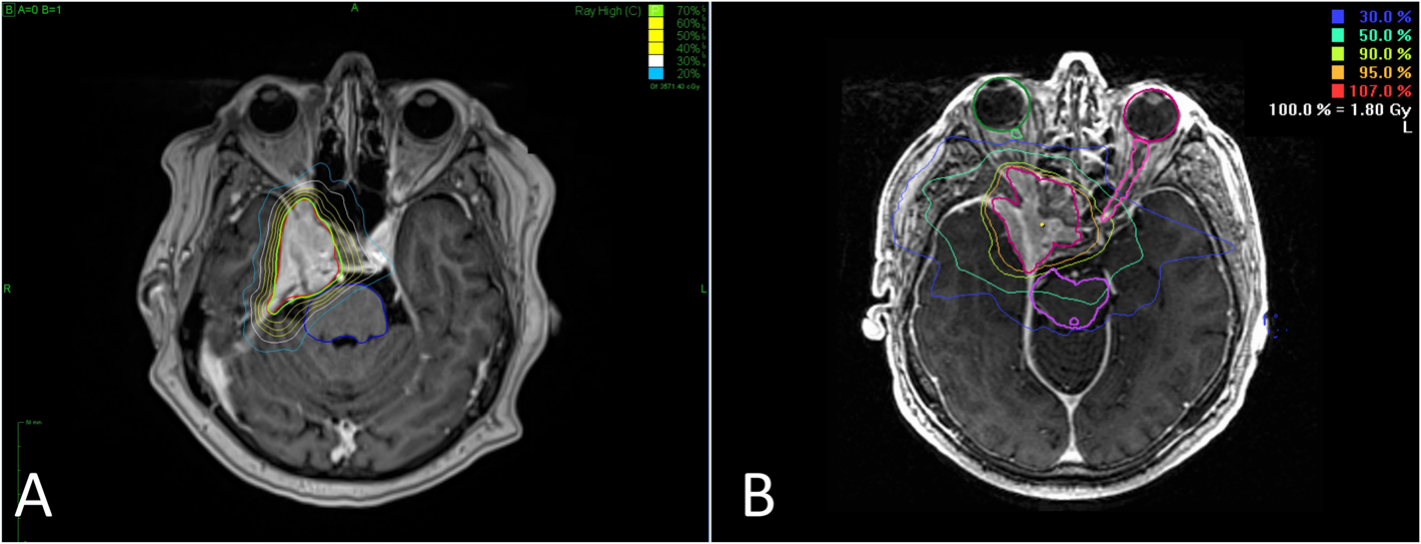
**a** Typical treatment plan for CK-hFSRT in a case of sphenoid wing meningioma. **b** Typical treatment plan of nFSRT for a sphenoid wing meningioma

Radiotherapy parameters for the two groups are shown in Table 2. Several fractionation regimens were used in both groups, the most common being 5 × 5 Gy in the CK-hFSRT group and 33 × 1.8 Gy in the nFSRT group. The mean GTV was significantly lower in the CK-hFSRT group than in the nFSRT group (10.1 ± 11.9 ccm vs. 25.1 ± 31.2 ccm, *p* < 0.001). In the CK-hFSRT group, the mean single dose was 5.2 ± 0.9 Gy, median dose per fraction was 5 Gy, mean prescribed total dose was 24.6 ± 4.9 Gy, and median prescribed total dose was 25 Gy (Fig. 1).

**Table 2:** Treatments and dosimetric features

	CK hFSRT	nFSRT	*p*-value
GTV [ml], mean ± sd	10.1 ± 11.9	25.1 ± 31.2	< .001
Single dose [Gy], mean ± sd	5.2 ± 0.9	1.8 ± 0.1	< .001
Single dose [Gy], median (range)	5.0 (2.67–8)	1.8 (1.8–2.8)	
Total dose [Gy], mean ± sd	24.6 ± 4.9	56.9 ± 4.2	< .001
Total dose [Gy], median (range)	25 (15–61)	59.4 (32.4–63)	
Prescription isodose [%], mean ± sd	75.6 ± 5.3	95 ± 0	< .001
Prescription isodose [%], median (range)	75.0 (60.0–100.0)	95 (95–95)	
EQD2 [Gy], mean ± sd	44.1 ± 8.1	54. 2 ± 3.8	< .001
EQD2 [Gy], median (range)	43.8 (26.3–100.0)	56.43 (30.78–60)	
BED [Gy], mean ± sd	88.1 ± 16.3	108.5 ± 7.5	< .001
BED [Gy], median (range)	87.5 (52.5–200)	112.9 (61.6–120)

In the nFSRT group, the mean single dose was 1.8 ± 0.1 Gy, median dose per fraction was 1.8 Gy, mean total dose was 56.9 ± 4.2 Gy, and median total dose was 59.4 Gy. The mean BED_2_ was significantly lower in the CK-hFSRT group (88.1 ± 16.3 vs. 108.5 ± 7.5, *p* < 0.001). The mean EQD2_2_ was also significantly lower in the CK-hFRST group (44.1 ± 8.1 Gy vs. 54. 2 ± 3.8 Gy, *p* < 0.001).

### LC

**Fig. 2:**
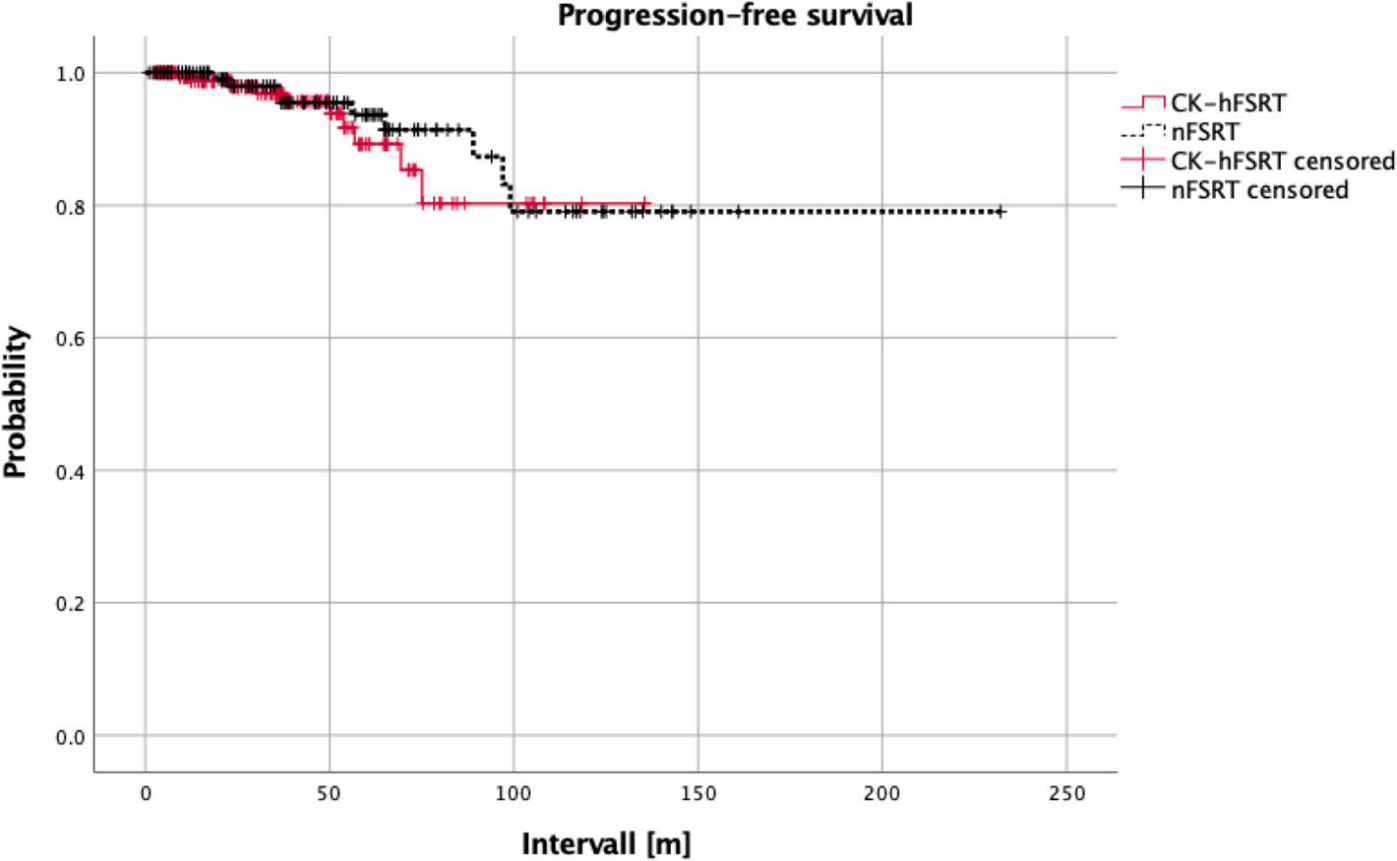
There were no significant differences in LC-rates between the nFSRT group and the CK-hFSRT group (*p* = 0.56, HR = 0.76, 95% CI, 0.3–1.9)

In the overall cohort, the 1-, 3-, 5-, and 10-year LC rates were 99.6, 97.3, 91.6, and 78.4%, respectively. There were no significant differences in LC rates between the nFSRT and CK-hFSRT groups (*p* = 0.56, hazard ratio [HR] = 0.76, 95% confidence interval [CI]: 0.3–1.9, Fig. 2). In the CK-hFSRT group, the 1-, 3-, and 10-year LC rates were 99.4, 96.8, and 80.3%, respectively. In the nFSRT group, the 1-, 3-, and 10-year LC rates were 100, 99, and 79.1%, respectively.

The largest volume treated in the CK-hFSRT group was 76.2 ccm. When only examining patients with a GTV ≤76.2 ccm in the nFSRT group (*n* = 92), there was still no significant difference with regard to LC-rates compared to the CK-hFSRT group (*p* = 0.17, HR = 0.44, 95% CI: 0.13–1.42).

**Fig. 3:**
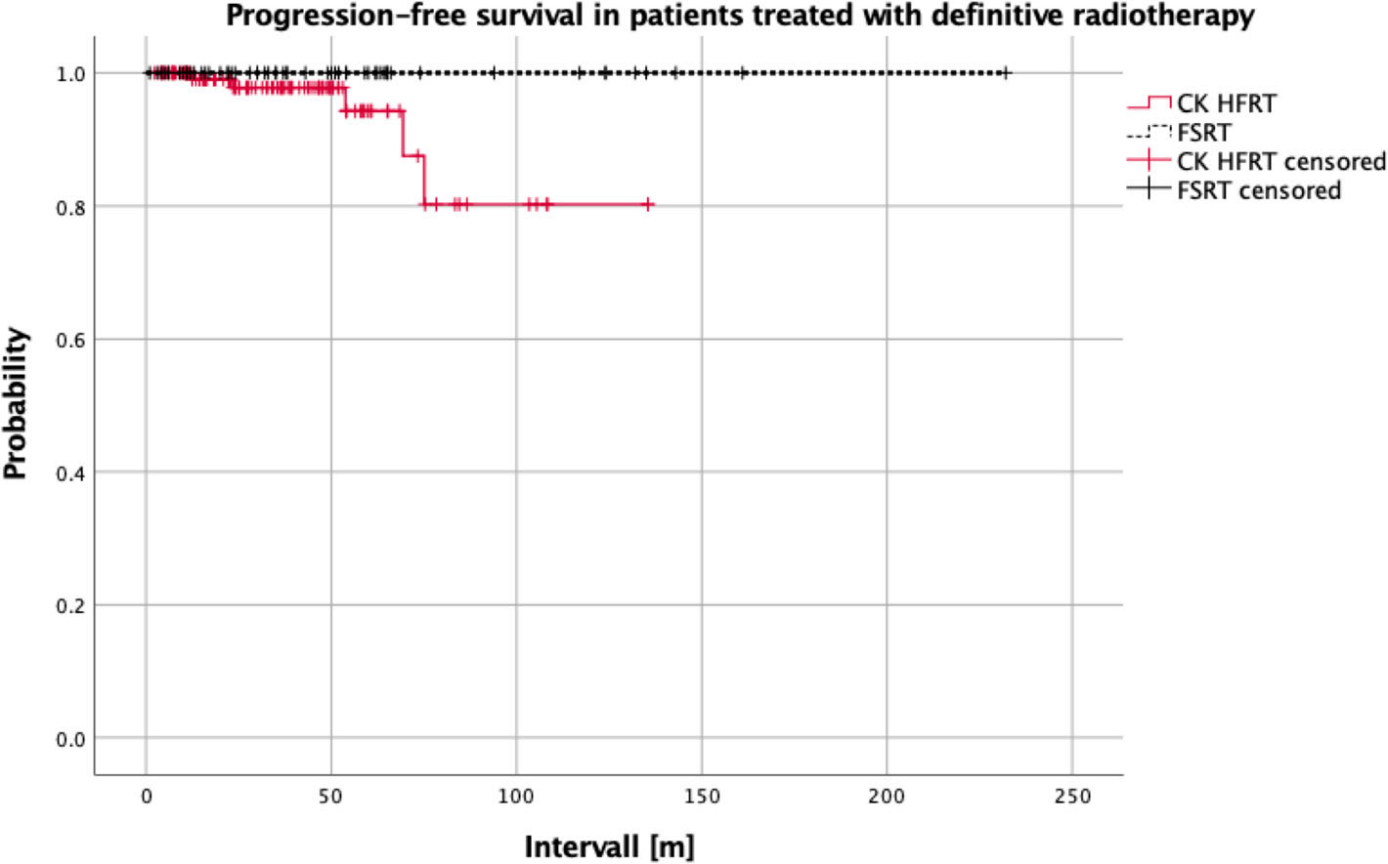
No relapses were seen in the cohort of patients treated with definitive nFSRT. However, due to the small number of patients with long term follow-up no significant differences were observed between the CK-hFSRT with definitive treatment and the nFSRT group with definitive treatment (*p* = 0.32, HR = 0.02, 95% CI, 0.0–40.2)

Interestingly no relapse was seen in the cohort of patients treated with definitive nFSRT (Fig. 3). Due to the relatively small number of patients with long term follow-up in the subgroups no significant differences were seen between the CK-hFSRT with definitive treatment and the nFSRT with definitive treatment groups (*p* = 0.32, HR = 0.02, 95% CI: 0.0–40.2).

Atypical meningioma were excluded from this analysis, even though a large group of patients the histological diagnosis was only supposed on the base of clinical behavior and imaging. Nevertheless, we did not find any correlation between patients for whom histology was available vs. no-histology group in terms of LC or progression free survival.

### Toxicity

Toxicity following RT was very low in both treatment groups. After CK-hFSRT a total of 16 patients (7.8%) had new mild (CTCAE I°) or moderate (CTCAE II°) cranial neuropathy. CTCAE II° trigeminal neuralgia was the most common cranial neuropathy (6.3%). Besides trigeminal neuralgia, cranial deficits in the CK-hFSRT group included one case (0.49%) of mild (CTCAE I°) visual disturbance in a patient with a clival lesion and one mild (CTCAE I°) case of third and sixth cranial nerve deficits in a patient with a spheno-petro-clival meningioma. One case (0.49%) of therapy-associated carotid artery occlusion was reported in a patient with a large spheno-petro-clival meningioma encasing the carotid artery, which resulted in a transient facial nerve (CTCAE III°) deficit. No new seizure onset was reported. in all patients who presented major complications the side effect was attributed to encasement of cranial nerves or vascular structures.

In the nFSRT treatment group, 12 patients (8.8%) had new mild or moderate (CTCAE ≤ II°) cranial neuropathy with four patients (2.9%) that developed mild (CTCAE I°) and one patient (0.7%) that developed moderate (CTCAE II°) optical pathway toxicity. Four patients (2.9%) had mild (CTCAE I°) and one patient (0.7%) had moderate (CTCAE II°) hearing impairment. One patient (0.7%) had mild and two patients (1.47%) had moderate (CTCAE II°) trigeminal neuralgia. Four patients (2.9%) developed mild (CTCAE I°) and one patient (0.7%) developed moderate (CTCAE II°) cranial nerve sensory deficits. One case (0.7%) of an acute grade III brain edema was seen and two cases (1.5%) of severe (CTCAE III°) vascular stenosis were seen both potentially linked to radiotherapy. Additionally, one patient (0.7%) developed new seizures (CTCAE II°).

## Discussion

We collected a large cohort of patients with skull base meningioma and provided a unique comparison between conventionally fractionated and hypofractionated radiotherapy approaches. This was done in order to shed light on the therapeutic potential of hFSRT in meningiomas that are not suitable for single fraction radiosurgery and are generally treated by nFSRT. Our retrospective analysis did not find a significant difference in LC-rates between the two groups (*p* = 0.56, HR = 0.76, 95% CI, 0.3–1.9). In general LC rates were satisfactory and comparable to those of single-fraction SRS or surgical treatment approaches [15–20]. Indeed, 10-year survival rates were close to 80% in both groups. Toxicities were limited and comparable between the groups suggesting a beneficial risk-benefit profile for both techniques in these patients.

Single-fraction SRS has been widely used as a treatment alternative to surgical resection, for small-to-moderate volume skull base meningiomas without close proximity to radiosensitive structures while the use of conventionally fractionated RT is more common in larger meningiomas that are not suitable for surgery [15–17, 21, 22]. LC rates in fractionated regimens range between 80 and 100%, depending on the size of the lesions, location, dose applied, and length of follow-up [4, 8, 23–37]. The results of several studies directly comparing the outcomes of nFSRT and SRS suggest that both are safe and effective techniques for treating skull base meningiomas and provide comparable satisfactory long-term tumor control [38–41]. In a multicenter pooled analysis of skull bases meningiomas Combs et al. compared 119 patients who received single fraction SRS to 808 patients who received nFSRT and showed significantly worse LC rates for the SRS treated patients using univariate analysis. This was also true when comparing only patients with smaller volumes (< 47.0 cc). However, these differences in LC were not seen after multivariate analysis [36]. The authors concluded that SRS is an alternative to nFSRT. However, following the Combs et al. study doubts remained in the neuro-oncology community regarding whether single fraction SRS is an adequate option for skull bases meningioma due to the lower LC rates for SRS in the univariate analysis.

According to current practice guidelines, the main criteria for selecting between FSRT and SRS are the average diameter of the meningioma, clearly recognizable tumor margins, and close proximity to radiation-sensitive structures. Effectiveness of SRS is seemingly reduced in larger tumors > 7.5 cc [42], and the risk-benefit ratio becomes unfavorable in these patients. Similarly, SRS is unfavorable in patients with tumors lying less than 3–5 mm away from radiosensitive structures, especially the anterior optic pathway. Some authors have therefore suggested the use of hFSRT for these cases, utilizing many of the same platforms used for SRS [10, 43–48]. In 2018, Meniai-Merzouki et al. revealed LC rates of 81% after 24 months in 96 patients who received CK-hFSRT as a primary treatment. This cohort included 57 patients who received definitive RT, ten patients who received adjuvant RT, and 29 patients who received RT after local progression after prior surgery alone [44]. These LC rates are a bit lower than the ones shown in the current study and reasons for this difference remains unclear.

Unger and colleagues reported on 173 patients with meningiomas where 56% underwent single fraction SRS with Gamma Knife, while the remaining patients received CK-hFSRT using two to five fractions [48]. The median dose for SRS was 15 Gy and the usual regimen was 25 Gy in five fractions for hFSRT. Two-year risk of symptomatic edema was 3.2% for multisession stereotactic radiation therapy, and 12.5% for SRS. Tumor size greater than 4.9 cc was also a significant predictor of symptomatic edema [48]. HFSRT has been typically adopted for the treatment of perioptic meningiomas. Midterm follow-up data on multisession radiosurgery of perioptic meningiomas have been reported [5, 45, 47, 49–53] with encouraging results showing tumor control rates > 90% with very limited toxicity to the anterior optic pathway. The authors were able to show that treatment of meningiomas in proximity or even encasing the optic nerve and chiasm by hFSRT is possible with a limited risk of radio-induced optic neuropathy that occurred only in 0–5% of cases [5, 47]. Nevertheless, especially in meningiomas in direct contact with the anterior optic pathway, the sensitivity of blood supply of the optical system must be considered; a vascular damage could lead to secondary late toxicity and thus loss of vision as result of high-dose fractions. On the other hand, a suboptimal dose can cause a visual function deterioration as a result of tumor progression [53]. If we look to our overall data, patients treated with hFSRT had mostly lesions with volumes ranging 8–25 cc or with a close relationship to critical structures, whereas nFSRT was adopted in larger tumors or in those encasing the optic apparatus. We can consider this a balanced indication for treatment strategy. Furthermore, in patients who presented major complications after hFSRT, these were attributed to encasement of or proximity to cranial nerves or vascular structures. Accordingly, due to the relative inhomogeneity of hFSRT as compared to nFSRT, encasement of critical structures, i.e. in optic nerve sheath meningiomas, should be considered as a factor in favor of nFSRT to avoid hot spots inside the GTV [54]. On the other hand, because of better conformal dose distribution, hFSRT would be a better choice in tumor adjacent to very sensitive OAR like perioptic meninigiomas.

A point deserving a comment is the difference of BED between nFSRT and hFSRT in this series. Indeed, nFSRT had a median BED of 113 Gy_2_ versus 87.5 Gy_2_ in hFSRT treatments. This can be explained on the base of a higher confidence with normofractionation about toxicity whereas a more conservative approach was adopted with a hypofractionated scheme. Notheworthy, the lower BED of hFSRT was sufficient to reach a similar level of LC suggesting that a similar BED is sufficient to warrant efficacy and confirming previous data [5, 45].

### Limitations

This study has several limitations. First, the retrospective approach is prone to bias, including potential cases where patients with relapse may seek treatment from a different healthcare provider, due to frustration with results of the previous treatment. Such instances may provide potential confounders that lead to the loss of follow-up data especially in patients that suffer from relapse. Second, the fractionation schedules and BEDs are heterogeneous within the two cohorts. Third, the cohorts are heterogeneous in such a way that they comprise patients who received radiotherapy in a definitive setting, both at primary diagnosis or at relapse and also in patients who received RT in an adjuvant setting.

Fourth, a significant subset of patients did not receive a histological verification of the diagnosis. Finally, some concerns remain about the very long-term toxicity of hFSRT – since these data are not available yet.

However, to our knowledge this is the first multicenter analysis comparing CK-hFSRT and nFSRT. We showed excellent LC and low side effects rates for patients treated with CK-hFSRT and nFSRT. The efficacy and safety, at least at the mid-term (i.e. 10 years), as well as convenience for patients is a strong argument for the use of CK-hFSRT in patients suffering from selected skull base meningioma.

## Conclusion

In summary, this analysis of pooled data from three centers showed excellent LC and low side effect rates for patients treated with CK-hFSRT or nFSRT. The efficacy, safety, and convenience of a shortened treatment period provide a compelling case for the use of CK-hFSRT in patients with skull base meningioma of moderate size and provided that OAR constraints are met.

## Data Availability

Data in the manuscript are available by contacting the corresponding author.
