# Correction to: Normofractionated stereotactic radiotherapy versus CyberKnife-based hypofractionation in skull base meningioma: a German and Italian pooled cohort analysis

**DOI:** 10.1186/s13014-020-01707-z

**Published:** 2020-12-14

**Authors:** Alfredo Conti, Carolin Senger, Güliz Acker, Anne Kluge, Antonio Pontoriero, Alberto Cacciola, Stefano Pergolizzi, Antonino Germanò, Harun Badakhshi, Markus Kufeld, Franziska Meinert, Phuong Nguyen, Franziska Loebel, Peter Vajkoczy, Volker Budach, David Kaul

**Affiliations:** 1 Department of Neurosurgery, University of Bologna, Bologna, Italy; 2 Department of Neurosurgery, Charité Universitätsmedizin Berlin, Berlin, Germany; 3 CyberKnife Center, Charité Universitätsmedizin Berlin, Berlin, Germany; 4 Department of Radiation Oncology, Charité Universitätsmedizin Berlin, Berlin, Germany; 5 Berlin Institute of Health (BIH), 10178 Berlin, Germany; 6 Department of Radiation Oncology, University of Messina, Messina, Italy; 7 Ernst Von Bergmann Medical Center, Department of Radiation Oncology, Potsdam, Germany

## Correction to: Conti et al. Radiation Oncology (2019) 14:201 10.1186/s13014-019-1397-7

Following publication of the original article [1], we have been notified that there is a mistake in the names of the authors. Correct names of the authors are provided below:

Alfredo Conti^1,2^, Carolin Senger^3,4^, Güliz Acker^2,3,5^, Anne Kluge^3,4^, Antonio Pontoriero^6^, Alberto Cacciola^6^, Stefano Pergolizzi^6^, Antonino Germanò^1^, Harun Badakhshi^7^, Markus Kufeld^3^, Franziska Meinert^2,3^, Phuong Nguyen^2,3^, Franziska Loebel^2,3^, Peter Vajkoczy^2,3^, Volker Budach^3,4^, David Kaul^4,*^
